# Postpartum Suicidality beyond Depression: a Systematic Review of Risk Profiles and Prevention Gaps

**DOI:** 10.1007/s11920-026-01706-z

**Published:** 2026-07-27

**Authors:** Valentina Baldini, Martina Gnazzo, Giorgia Varallo, Diana De Ronchi, Marco Carotenuto, Andrea Fiorillo

**Affiliations:** 1 Department of Biomedical and Neuromotor Sciences, University of Bologna, Bologna, Italy; 2 Department of Biomedical, Metabolic and Neural Sciences, University of Modena and Reggio Emilia, Modena, Italy; 3 Clinic of Child and Adolescent Neuropsychiatry, Department of Mental Health, Physical and Preventive-Medicine, University of Campania “Luigi Vanvitelli”, Caserta, Italy; 4 Department of Psychiatry, University of Campania “Luigi Vanvitelli”, Naples, Italy

**Keywords:** Postpartum, Suicidal ideation, Maternal suicide, Perinatal mental health

## Abstract

**Purpose of Review:**

Suicidal ideation and behavior during the postpartum period are increasingly recognized as important contributors to maternal morbidity and mortality, particularly in high-income countries. Although postpartum depression is a well-established risk factor, emerging evidence suggests that suicidality may also occur independently and therefore remain underrecognized in clinical practice. This systematic review aimed to synthesize current evidence regarding the prevalence of suicidal ideation and behavior in the postpartum period and to identify associated risk factors.

**Recent Findings:**

A systematic search of PubMed, Web of Science, EMBASE, and PsycINFO was conducted up to March 2025 following PRISMA guidelines. Twenty studies met inclusion criteria, encompassing 352,726 postpartum women across diverse international settings. Reported prevalence of suicidal ideation ranged from 2.2% to over 50%, depending on the population studied and assessment methods used. The most frequently identified risk factors included postpartum depression, anxiety, trauma history, intimate partner violence, unplanned pregnancy, low socioeconomic status, low social support, and adverse childhood experiences. Notably, several studies reported suicidal ideation even in the absence of clinical depression. Only a limited number of studies addressed preventive interventions.

**Summary:**

Postpartum suicidality appears to be a multifactorial phenomenon that cannot be explained solely by depressive symptomatology. The findings highlight the need for broader screening strategies that incorporate psychosocial and trauma-related factors in addition to depression. Integrated and trauma-informed approaches to screening and prevention may improve early identification of at-risk individuals. Future research should prioritize longitudinal designs and culturally sensitive investigations to better inform prevention and intervention strategies.

## Introduction

Suicide has become one of the leading causes of maternal mortality in high-income countries, accounting for up to 20% of postpartum deaths [27, 36, 37], particularly during the late postpartum period (defined here as after six weeks and up to 18 months after childbirth), when routine medical and psychiatric monitoring often decreases [18, 25]. Notably, higher rates of suicide are observed in female individuals with psychiatric histories [15]. Indeed, the postpartum period is a time of significant biological, psychological, and social adaptation, and it is characterized by increased vulnerability to psychiatric disorders such as depression, anxiety, and psychosis [3]. One of the most severe outcomes of perinatal psychopathology—spanning both the antenatal and postpartum periods—is suicidal ideation and behavior, which are critical public health challenges [2, 35].

Despite its clinical significance, it is challenging to make an accurate estimation of the actual burden of suicidal ideation and suicidal behavior during the postpartum period. Epidemiological prevalence rates range from 3 to 14%, depending on the studied population, the screening instruments used, and the point of assessment [19, 40]. Among high-risk groups—such as female individuals with postpartum depression, a history of trauma, or lacking adequate support—the prevalence may exceed 25% [32]. Suicide attempts during the perinatal period are less common but often involve highly lethal means and result in more severe medical outcomes compared to non-perinatal attempts [43].

Several biopsychosocial risk factors are linked to an increased risk of suicidal ideation and behaviors during the postpartum period. These factors include a history of psychiatric illness (e.g., depression, bipolar disorder, and borderline personality disorder), along with a personal or family history of suicide, substance use, sleep disturbances, socioeconomic challenges, and intimate partner violence [6, 55]. Recent studies have also emphasized the influence of perinatal-specific stressors such as unplanned pregnancy, birth trauma, breastfeeding difficulties, and feelings of maternal inadequacy [1, 4, 24, 28].

Importantly, emerging evidence suggests that suicidal ideation during the postpartum period may not always be secondary to depressive symptoms [49]. Some individuals may experience intrusive or ego-dystonic thoughts of self-harm even when diagnosable depression is absent, highlighting the need for comprehensive and nuanced assessment tools [12]. Furthermore, the stigma surrounding maternal mental health can inhibit help-seeking behaviors, leading to underreporting and missed opportunities for intervention [14].

There are still few and unevenly implemented preventive measures to address postpartum suicidality across various healthcare systems. Although universal screening for depression and suicidal thoughts throughout pregnancy and the postpartum period is recommended by several professional organizations, its application in clinical practice remains inconsistent [57]. Promising interventions include stepped-care models that integrate obstetric and mental health services, home-visiting programs, trauma-informed psychotherapy, and peer support initiatives [29]. Nevertheless, there is an urgent need for more robust evidence concerning the effectiveness and scalability of these approaches in specifically reducing suicidal ideation and behaviors.

In light of these concerns, this systematic review aims to synthesize current evidence regarding the prevalence and risk factors associated with suicidal ideation and behavior during the postpartum period. By consolidating findings across diverse populations and methodologies, this review seeks to inform clinical guidelines, identify knowledge gaps, and support the development of targeted interventions for suicide prevention among postpartum individuals.

## Methods

This systematic literature review was conducted and reported according to the preferred reporting items for systematic reviews and meta-analysis PRISMA guidelines [41]. The study protocol was registered in advance on PROSPERO (CRD420251017138).

### Search Strategy

A systematic search was conducted from inception until March 2025 on PubMed, Web of Science, EMBASE, and PsycINFO using the following search criteria: ("postpartum period"[MeSH Terms] OR postpartum OR postnatal OR perinatal OR "after childbirth" OR puerperium) AND ("suicide"[MeSH Terms] OR "suicidal ideation"[MeSH Terms] OR suicidality OR "suicidal thoughts" OR "suicide attempt" OR "self-harm") AND ("risk factors"[MeSH Terms] OR "Depression, Postpartum"[MeSH Terms] OR "mental disorders"[MeSH Terms] OR "psychiatric status rating scales"[MeSH Terms] OR "social support"[MeSH Terms] OR prevention OR screening OR intervention OR "predictive factors" OR prevalence). The search was conducted from inception rather than limited to recent years, given that postpartum suicidality as a distinct research focus has only emerged systematically over the past two decades and a restricted timeframe would have precluded the inclusion of foundational studies essential for a comprehensive synthesis.

### Eligibility Criteria

Studies were eligible for inclusion if they met the following criteria: (1) original, peer-reviewed research articles; (2) reported on suicidal ideation, suicidal behaviors including suicide attempts, or completed suicide occurring during the postpartum period (up to 18 months after childbirth); (3) included human participants aged 18 years or older; and (4) employed validated tools or structured methods to assess suicidality.

It should be noted that suicidal ideation, suicide attempts, and death by suicide are related but conceptually and clinically distinct constructs, with increasing proximity to mortality risk. Although all three were eligible for inclusion, most included studies focused on suicidal ideation, with fewer studies specifically designed to examine suicide attempts or completed suicide. This outcome heterogeneity should be considered when interpreting the synthesized findings.

We included studies with various designs, such as cross-sectional, cohort, and case–control studies. Both community-based and clinical samples were taken into account. Studies were excluded if they: (1) focused exclusively on the antenatal period without reporting postpartum data; (2) were review articles, editorials, commentaries, or conference abstracts; (3) failed to report specific outcomes related to suicidal ideation or behavior; or (4) were not published in English.

Eligibility assessments were conducted by two independent reviewers (V.B. and M.G.) based on predetermined inclusion and exclusion criteria. Any disagreements were resolved through discussion or consultation with a third reviewer (G.V.).

### Selection of the Studies

All relevant original research articles were identified based on their titles and abstracts during the screening phase. At this stage, articles lacking information, including those not in English, reviews, case reports/series, conference abstracts, editorials, and viewpoints, were excluded. The full texts of the articles identified in the screening phase were reviewed to determine whether they met the selection criteria. These full texts were also manually searched to find additional studies. Two reviewers (VB and MG) conducted the double-blind selection. No sex or age criteria were used for article eligibility.

### Data Extraction

Data from each included study were extracted using a standardized form developed a priori by the research team. For each study, we collected details on the authors, year of publication, country, study design, and sample size. We also documented the instruments used to assess suicidal ideation, suicide attempts, or suicide deaths, along with the reported prevalence or rate of suicidal thoughts or behaviors during the postpartum period.

Additionally, we extracted information on the specific postpartum timeframe when suicidal ideation or behaviors were assessed (for instance, at six weeks, three months, or within the first year following childbirth), as well as the primary risk factors identified by each study. These factors included psychological, social, demographic, or clinical variables associated with an increased risk of postpartum suicidality. When available, we also incorporated relevant statistical data, such as odds ratios or confidence intervals, along with any details on protective factors or preventive interventions mentioned.

Two reviewers independently conducted the data extraction (V.B. and M.G.). Any discrepancies were resolved through discussion or, if necessary, consultation with a third reviewer (A.F.). For studies reporting on multiple time points or outcomes, we selected the most relevant postpartum data in accordance with the inclusion criteria established for this review.

### Critical Appraisal Assessment

Included studies were evaluated with the Newcastle–Ottawa Scale (NOS), which assesses the risk of bias in observational studies on three domains (selection, comparability, and exposure) and provides an overall score ranging from 1 (highest risk of bias) to 9 (lowest risk of bias) [22]. To reduce the selection bias arising from included studies and the bias in rating the quality of studies, these procedures were initially carried out independently by the two authors (V.B. and M.G.). Any discrepancies were resolved by joint consensus following the independent evaluations.

## Results

### Flow Chart of Included Studies

The database search identified 3,117 records: 968 from PubMed, 779 from Web of Science, 950 from EMBASE, and 420 from PsycINFO. After removing 1,000 duplicates, 2,117 unique records remained and were screened based on their titles and abstracts. Of these, 1,750 records were excluded for not meeting the inclusion criteria.

The full texts of 367 articles were assessed for eligibility. Following the full-text review, 347 articles were excluded for the following reasons: population not in the postpartum period (*n* = 142), outcomes unrelated to suicidal ideation or behavior (*n* = 96), insufficient data (*n* = 59), and ineligible article types (e.g., review or editorial) (*n* = 50).

**Fig. 1:**
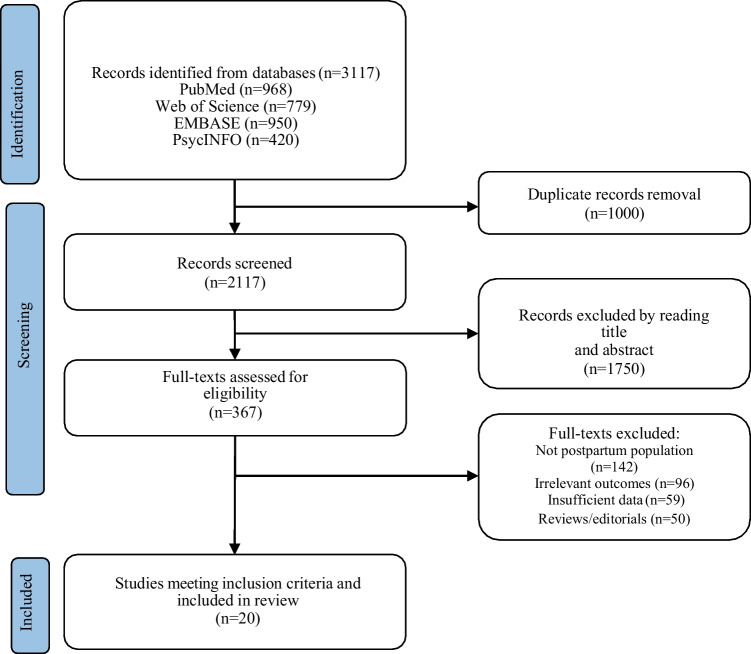
Flow-chart describing the study selection process

Ultimately, 20 studies met all inclusion criteria and were included in the qualitative synthesis [7, 10, 11, 13, 17, 21, 26, 30, 34, 42, 44–48, 50–54]. A flow diagram illustrating the selection process is provided in Fig. 1.

### Ratings of Study Quality and Risk of Bias

Based on the study’s quality ratings, 12 studies were rated as good quality [10, 11, 17, 21, 26, 30, 42, 46–48, 52, 53] 6 as moderate quality [7, 13, 34, 45, 50, 54], and 2 as poor quality [44, 51]. Since most of the studies included in this review were of moderate or good quality, the risk of bias from these was low. Two researchers independently extracted data on the variables from the studies.

### Characteristics of the Included Studies

The qualitative synthesis included 20 studies encompassing 352,726 women. These studies were published between 2006 and 2025 and conducted in various countries, including the United States, Canada, Brazil, Spain, Ethiopia, South Korea, Pakistan, Taiwan, and Italy, reflecting a diverse geographic representation.

Most studies utilized a cross-sectional design, while others employed cohort or case–control methodologies. Sample sizes varied significantly, ranging from 15 to over 327,279 participants, depending on the study design and data source. Suicidal ideation and behavior were assessed using various standardized instruments, including single items from the Edinburgh-Postnatal-Depression-Scale (EPDS) or Patient Health Questionnaire-9 (PHQ-9), as well as structured interviews like the C-SSRS (Columbia-Suicide Severity Rating Scale).

The postpartum timeframe examined in the studies ranged from 48 h to 18 months after childbirth, with many focusing on the first 6 to 12 months postpartum. The reported prevalence of suicidal ideation varied considerably, from as low as 2.2% to over 50%, depending on the measurement tools, populations studied, and contextual factors. Several studies also reported on suicide attempts and completed suicides, particularly those using national health records or hospital data.

A wide range of risk factors was identified, including postpartum depression, anxiety, intimate partner violence, low socioeconomic status, poor social support, unplanned pregnancy, a history of trauma, and psychiatric comorbidities. Notably, some studies highlighted suicidal ideation even in the absence of diagnosable depression, suggesting the need for more comprehensive screening tools.

Table 1 and 2 summarizes the characteristics of the included studies, such as study design, population, instruments used, prevalence of suicidal ideation or behavior, and identified risk factors.

**Table 1:** Characteristics of included studies. NR = Not reported

Author, Year	Country	Study design	Sample size	Mean age (M ± SD)	Quality assessment
Belete, 2019	Ethiopia	Cross-sectional	988	NR	Moderate
Bodnar-Deren, 2016	USA	Cross-sectional	1073	30.17 ± 6.18	Good
Boisvert, 2023	Canada	Cohort	216	33 ± 1.5	Good
Comtois, 2008	USA	Case–control	1770	NR	Moderate
Faisal-Cury, 2021	Brazil	Cross-sectional	517	26.5 ± 7	Good
Gold, 2011	USA	Cross-sectional	2083	32 ± 11	Good
Lee, 2024	South Korea	Cross-sectional	1496	NR	Good
Martínez-Galiano, 2025	Spain	Cross-sectional	1114	33.3 ± 3.96	Good
Muzik, 2016	USA	Cohort	116	29.5 ± 5.93	Moderate
Paris, 2009	USA	Cohort	32	32.5 ± 5.6	Good
Schafer, 2024	USA	Cross-sectional	234	30.19 ± 5.13	Low
Schiff, 2006	USA	Case–control	2724	NR	Moderate
Sit, 2015	USA	Cohort	628	28.7 ± 5.2	Good
Stefana, 2024	Italy	Cross-sectional	3850	33.2 ± 5.1	Good
Szpunar, 2020	USA	Cohort	15	31.6 ± 2.1	Good
Tabb, 2018	Brazil	Cross-sectional	701	NR	Moderate
Tavares, 2011	Brazil	Cross-sectional	1053	NR	Low
Vigod, 2019	Canada	Cohort	327,279	30.5 ± 5.2	Good
Weng, 2018	Taiwan	Case–control	5308	NR	Moderate
Weng, 2016	Taiwan	Case–control	1529	NR	Good

**Table 2:** Overview of prevalence, timing, and risk factors for suicidal ideation and behavior in the postpartum period

Author, year	Measures of suicidal ideation/behavior	Prevalence or rate of suicidal ideation/behavior (%)	Timeframe postpartum	Identified risk factors
Belete, 2019	Suicidal behaviour (ideation, plan, or attempt via MINI)	78.3% ideation; 72.5% plan; 36.2% attempt	6 weeks	Low education; verbal abuse; history of rape; unplanned pregnancy; sick infant health status
Bodnar-Deren, 2016	Suicidal ideation measure with item 9 of PHQ-9 and Item 10 of EPDS	2.2%	48 h	Antepartum complication; low education; baseline depression and anxiety
Boisvert, 2023	Suicidal thoughts measured with Item 10 of EPDS	7.87%	3 months	Pre-existing anxiety and/or depression; negative coping strategies
Comtois, 2008	Number of suicide attempts	1 in 2,277 women were hospitalized for a suicide attempt postpartum	12 months	Prior psychiatric hospitalization; substance use disorder; dual diagnosis
Faisal-Cury, 2021	Suicidal ideation measure with item 9 of PHQ-9	10.3%	6 months	Postpartum depression; low education; low income; not being married
Gold, 2011	Suicide deaths	3.1%	12 months	Current depressed mood; intimate partner problems
Lee, 2024	P4 Suicidal screener	11.2%	12 months	Domestic violence; past depressive disorder; unplanned pregnancy; postpartum depression
Martínez-Galiano, 2025	Suicidal ideation measured with CARE-MQ	12%	6 months	Low income level; unplanned pregnancy; problems during childbirth; ICU admission; hospital readmission
Muzik, 2016	Suicidal ideation measured with Item 5 of PDSS	25%	18 months	Maltreatment-related shame; lower resilience; not being married; lower family support
Paris, 2009	Suicidal ideation measured with PDSS	53% high suicidality (PDSS score ≥ 12)	4 months	Greater postpartum depression severity; higher anxiety, confusion, guilt, emotional lability; lower maternal self-esteem and preparedness for motherhood; higher parenting stress; distorted perceptions of infant
Schafer, 2024	Suicidal ideation measure with item 9 of PHQ-9	3.85%	2 months	Antenatal anxiety
Schiff, 2006	Number of suicide attempts	43.9 per 100,000 live births (520 attempts over 1.18 million births)	12 months	Fetal or infant death; prenatal smoking; early or no prenatal care; low education, single, young maternal age
Sit, 2015	Suicidal ideation measured with Item 10 of EPDS	5.4%	6 weeks	Childhood physical abuse; anxiety symptoms; earlier depression onset; lower functioning; psychiatric comorbidities
Stefana, 2024	Suicidal ideation measured with Item 10 of EPDS	17%	12 months	NR
Szpunar, 2020	Suicidal ideation (C-SSRS)	3.6%	6 weeks	Lifetime suicide attempts; depression severity linked to lifetime suicidal ideation intensity postpartum
Tabb, 2018	Suicidal ideation (CIS-R single-item)	4%	12 months	Intimate partner violence; low social support; low income; prior psychiatric treatment
Tavares, 2011	Suicide risk (MINI suicidality module)	11.5%	3 months	Comorbidity of depression and anxiety greatly increases suicide risk
Vigod, 2019	Self-inflicted injury (including suicide attempts and deaths)	1.27 per 1000	12 months	NR
Weng, 2018	Number of attempted and completed suicide (hospital records and death registry)	Attempted suicide after miscarriage: aOR = 2.1 Attempted suicide after termination: aOR = 2.5 Completed suicide after stillbirth: aOR = 5.2 Completed suicide after miscarriage: aOR = 3.81 Completed suicide after termination: aOR = 3.12	12 months	Fetal loss (miscarriage, termination, stillbirth); mood disorder; anxiety; schizophrenia; substance use disorder; suicide history; being unmarried
Weng, 2016	Number of suicide attempts and completed suicides	Attempted: 9.91 per 100,000 Completed: 6.86 per 100,000	12 months	Attempted suicide: never married; caesarean delivery; history of suicide; postpartum depression Completed suicide: never married; low birth weight

*MINI* Mini-international neuropsychiatry interview; *PHQ-9* Patient health questionnaire-9; *EPDS* Edinburgh-postnatal-depression-scale; *PDSS* Panic disorder severity scale; *C-SSRS* Columbia-suicide severity rating scale; *CIS-R* Clinical interview schedule revise; *aOR* Adjusted odd ratio

## Narrative Synthesis

### Social-Demographic and Economic Risk Factors

Numerous studies have consistently reported low socioeconomic status as a factor linked to suicidality during the postpartum period. Educational level has been associated with suicidal ideation and attempts [7, 10, 17, 45], along with low income [17, 30, 50]. Another common factor identified was being unmarried [17, 34, 45, 54]. Young maternal age has been recognized as a factor that impacts vulnerability [45].

### Psychiatric History and Co-Morbidities

A psychiatric history, particularly involving depression and anxiety, is one of the strongest predictors of suicidality. Several studies have reported that postpartum suicidality is more prevalent among women with a psychiatric history of depression or anxiety [11, 26, 51]. Comorbid psychiatric diagnoses and psychiatric hospitalization prior to childbirth are especially significant in increasing the risk of suicidal behaviors [13]. Past suicide attempts have been associated with an increased risk during the postpartum period [48, 54].

### Psychological and Emotional Factors

Psychological vulnerabilities significantly contribute to suicide risk. The severity of depression acted as a consistent predictor [42, 48]. Additional psychological correlates included anxiety, confusion, guilt, emotional dysregulation, and low maternal self-esteem [42]. Shame related to maltreatment, low resilience, and negative perceptions of the infant also elevated mothers' risk for suicidality [34, 42]. Emotional dysregulation and negative coping strategies were also recognized as risk factors [11].

### Reproductive and Perinatal Health-Related Risk Factors

Challenges during pregnancy or delivery, along with complications during postpartum recovery, were also linked to increased suicidality. Antepartum pregnancy complications [10], ICU admissions or hospital readmissions [30], and the loss of a fetus or infant [45, 54] were all tied to higher rates of suicidal ideation and behavior. Regarding fetal loss, mothers who experienced miscarriages, stillbirths, or clinical terminations faced a significantly higher risk of death by suicide and suicide attempts [54]. Additionally, cesarean delivery appeared to be a risk factor for suicide attempts [53].

### Interpersonal and Social Risk Factors

Significant associations were observed with intimate partner violence and/or relationship problems (e.g., [21, 26, 50]). Verbal abuse (e.g., [7]), being unmarried (e.g., [17, 34]), and low family or partner support (e.g., [34]) were among the most common risk factors identified across studies. Childhood physical abuse was also noted as a significant early life stressor that predisposed individuals to suicidality [46].

### Obstetric and Infant-Related Risk Factors

Unplanned pregnancy has been identified in several studies as a risk factor linked to suicidal ideation (e.g., [7, 26, 30]). Infant-related risk factors and concerns have also surfaced, being indirectly documented regarding perceived infant health problems (e.g., [7]) and inaccurate maternal evaluations of the infant (e.g., [42]). Additionally, risks associated with childbirth were compounded by concerns or problems encountered during labor (e.g., [30]).

### Health Behavior and Care Access

Certain behaviors can influence the risk or severity of suicidality, including tobacco use during prenatal care (e.g., [45]), inadequate or delayed prenatal care (e.g., [45]), and failure to receive mental health treatment (indicated by unresolved psychiatric issues noted in various studies). Additionally, a history of substance use disorder (e.g., [13, 54]) has been associated with an increased risk of postpartum suicide.

## Discussion

To our knowledge, this represents the first systematic review specifically focused on suicidal ideation and behavior during the postpartum period. Our findings highlight significant variability in the reported prevalence of suicidal ideation, which ranges from 2.2% to over 50%, influenced by study design, assessment tools, population characteristics, and timing of evaluation. These results align with previous research indicating considerable heterogeneity in perinatal mental health assessments [16, 39].

Although postpartum depression is a well-established risk factor, our findings highlight that suicidal ideation may also occur independently of clinical depression. Specifically, our review identified a wide range of risk factors linked to postpartum suicidal ideation and behavior, categorized into three primary domains: sociodemographic, social, and clinical.

Sociodemographic factors seem to increase vulnerability to suicidal ideation and behavior during the postpartum period, including low educational attainment, low income, young maternal age, and being unmarried. These findings align with previous literature indicating that women facing socioeconomic disadvantages are more likely to experience mental health challenges during the perinatal period. A systematic review by Howard et al. revealed that low income and lower levels of education were among the strongest predictors of perinatal mental disorders, including suicidal ideation [23]. Furthermore, maternal age and relationship status have been linked to suicidality; younger and single mothers often report higher levels of psychological distress, possibly due to limited social support and greater economic stress [36, 37]. These consistent associations underscore the role of structural determinants in shaping maternal mental health outcomes and emphasize the need for targeted interventions that address social and economic vulnerabilities.

In addition to sociodemographic factors, our review identified several key social determinants consistently associated with suicidal ideation and behavior during the postpartum period. These included intimate partner violence, lack of social support, verbal abuse, and unplanned pregnancy. Intimate partner violence emerged as one of the most recurrent and potent social predictors, highlighting the critical intersection between interpersonal trauma and mental health [5]. These findings are supported by existing literature, such as a meta-analysis by Beydoun and colleagues that found a strong association between included intimate partner violence and suicidal ideation among perinatal women [9]. Moreover, a lack of perceived social support has been shown to exacerbate emotional distress during the postpartum period, increasing vulnerability to suicidal thoughts, particularly among women with concurrent depressive or anxiety symptoms [33]. Unplanned pregnancy, frequently linked to increased psychological stress and reduced coping capacity, also emerged as a relevant contributor to suicidality, in line with studies indicating poorer mental health outcomes among women with unintended pregnancies [20]. These findings emphasize the urgent need to address social vulnerabilities within routine postpartum care, including systematic screening for intimate partner violence and enhancing support networks.

The convergence of these factors highlights the multifactorial nature of postpartum suicidality, which cannot be reduced to a single cause but rather arises from the interaction of individual vulnerability, relational stress, and structural adversity. These insights emphasize the need for comprehensive screening strategies and holistic interventions that address both psychological symptoms and the broader psychosocial context of postpartum women.

Beyond sociodemographic and social determinants, clinical risk factors are a critical yet underexplored dimension of postpartum suicidality that warrants greater attention in both research and practice. Among the strongest clinical predictors identified in this review is a history of suicidal ideation or behavior. Consistent with established models of suicide risk, individuals with prior suicidal thoughts or attempts are at substantially elevated risk during the postpartum period [48, 54]. This finding underscores the importance of routinely and systematically inquiring about lifetime suicidal history during perinatal care, rather than limiting assessment to current symptoms. Importantly, this risk persists even among women who appear clinically stable or do not meet diagnostic criteria for a current psychiatric disorder, highlighting the need for longitudinal monitoring across the entire postpartum window.

A further clinical concern is the limitations of current screening practices. The most widely used perinatal screening tools—such as the Edinburgh Postnatal Depression Scale (EPDS) and the Patient Health Questionnaire-9 (PHQ-9)—were primarily designed to detect depressive symptoms, and their suicide-related items are often limited to a single question assessing self-harm ideation [31, 32]. While these instruments have demonstrated utility in identifying women at risk for postpartum depression, they may not adequately capture the full spectrum of suicidal ideation, particularly among women who experience suicidal thoughts without clinical depression—a pattern documented by several studies included in this review [12, 49]. Incorporating validated suicide-specific measures, such as the Columbia Suicide Severity Rating Scale (C-SSRS) or the Suicidal Ideation Attributes Scale (SIDAS), into routine postpartum care could substantially improve early detection and triage of at-risk individuals.

Finally, the role of transdiagnostic symptoms—those that cut across diagnostic categories and are not adequately captured by disorder-specific screening—deserves greater clinical attention. Symptoms such as hopelessness, insomnia, agitation, and emotional dysregulation have been identified as potent, modifiable risk factors for suicidality in the broader psychiatric literature [8, 56], and several studies in this review documented their relevance in postpartum populations [2, 11, 42]. Crucially, these symptoms may be present—and clinically actionable—even when a woman does not fulfill diagnostic criteria for a depressive or anxiety disorder. A transdiagnostic approach to assessment and intervention, targeting these modifiable symptom dimensions, may therefore offer a more sensitive and pragmatic framework for suicide prevention in postpartum care than one relying solely on diagnostic categories.

The stigma surrounding maternal mental health continues to be a significant barrier to seeking help during the postpartum period. Many women experiencing suicidal ideation hesitate to disclose their thoughts due to fear of being judged as unfit mothers, facing social rejection, or potentially losing custody of their children. These fears are often amplified by cultural narratives that idealize motherhood and discourage the expression of emotional vulnerability. Consequently, women may feel isolated and ashamed, contributing to the underreporting of symptoms and delays in accessing care when timely intervention is critical. In certain contexts, this stigma is reinforced by healthcare providers who may lack training in empathetic and nonjudgmental communication, further discouraging disclosure. A study by Oladeji and colleagues emphasized that stigma, when combined with low mental health literacy, significantly impairs treatment engagement among perinatal women [38]. Their findings suggest that many women either do not recognize their symptoms as clinically relevant or do not believe that effective help is accessible. Addressing this dual challenge of stigma and knowledge gaps is essential. Public health campaigns, provider education, and peer support interventions may play a key role in creating a more accepting and informed environment that empowers women to seek help without fear of repercussions.

This review has several limitations. Firstly, most of the included studies were cross-sectional, which limits the ability to draw causal inferences about the relationship between risk factors and suicidality. Secondly, the variation in the assessment tools used to measure suicidal ideation and behavior—ranging from individual items on screening questionnaires to structured clinical interviews—hinders direct comparability across the studies. Thirdly, cultural and regional differences may have affected both the reporting and prevalence estimates, particularly in contexts where stigma surrounding mental health is prevalent.

Fourthly, a notable limitation of this review concerns the heterogeneity of suicidality outcomes across included studies. Suicidal ideation, suicide attempts, and death by suicide are related but distinct constructs that differ substantially in clinical implications and proximity to mortality risk. Suicidal behavior—and especially completed suicide—represents a more proximal indicator of fatal outcome than ideation alone. Yet the majority of included studies assessed suicidal ideation using single items from depression screening tools (e.g., EPDS, PHQ-9), which are not specifically designed to capture the full severity spectrum of suicidal risk. Only a minority of studies employed structured clinical interviews or administrative data capable of identifying suicide attempts or completed suicides. Furthermore, it remains unclear whether any included study was specifically designed to identify risk factors for suicide death as a primary outcome, as opposed to ideation or attempts. This limits the extent to which findings can be directly extrapolated to mortality risk and underscores the need for future studies employing more granular and outcome-specific methodologies.

Finally, the limited availability and cross-study comparability of quantitative effect sizes precluded a more precise characterization of the relative strength of associations with individual risk factors.

In summary, postpartum suicidal ideation and behavior are complex and multifaceted issues influenced by a range of psychological, social, and structural factors. There is an urgent need for comprehensive screening protocols, stigma reduction initiatives, and well-evaluated interventions to better support at-risk individuals. Notably, variables such as parity — which may differentially influence maternal identity, social support, and psychological adaptation in first-time versus multiparous mothers — were not examined in any included study, representing a further gap in the literature that warrants future investigation.

Future research should prioritize longitudinal, culturally sensitive studies and thorough evaluations of preventive strategies.

## Conclusion

This systematic review is the first to concentrate exclusively on suicidal ideation and behavior during the postpartum period. The findings highlight the urgent necessity to incorporate suicidality screening into standard postpartum care to improve early detection and reduce preventable maternal deaths.

A wide range of psychological, social, and contextual factors—such as anxiety, trauma history, intimate partner violence, low social support, and unplanned pregnancy—are consistently associated with an increased risk. However, despite growing awareness, preventive strategies remain underdeveloped, inconsistently applied, and poorly evaluated across various settings.

There is an urgent need for universal screening protocols that extend beyond depression alone, along with the implementation of integrated, trauma-informed care pathways tailored for the postpartum population. Future research should focus on longitudinal and culturally diverse populations, rigorously evaluating the effectiveness of both preventive and therapeutic interventions.

Ultimately, tackling postpartum suicidality necessitates a comprehensive, multidisciplinary approach that integrates clinical vigilance, social support, and responsive health systems to guarantee early identification, timely intervention, and improved outcomes for new mothers.

## Key References

- Arditi-Arbel B, Hamdan S, Winterman M, Gvion Y. Suicidal ideation and behavior among perinatal women and their association with sleep disturbances, medical conditions, and known risk factors. Front Psychiatry. 2023;13:987673.
  - ○ This recent study highlights the complex interplay between sleep disturbances, medical comorbidities, and psychosocial stressors in perinatal suicidality. It contributes to the growing recognition that suicidal ideation in the perinatal period may arise from multiple interacting biological and psychosocial vulnerabilities.
- Chen C, Okubo R, Okawa S, et al. The prevalence and risk factors of suicidal ideation in women with and without postpartum depression. J Affect Disord. 2023;340:427–434.
  - ○ This study is particularly important because it demonstrates that suicidal ideation can occur independently of postpartum depression. The findings support the need for broader screening strategies that assess suicidality beyond depressive symptoms alone.
- Schafer K, Mulligan E, Shapiro MO, et al. Antenatal anxiety symptoms outperform antenatal depression symptoms and suicidal ideation as a risk factor for postpartum suicidal ideation. Anxiety Stress Coping. 2024.
  - ○ This paper provides evidence that antenatal anxiety may be a stronger predictor of postpartum suicidal ideation than depressive symptoms. It underscores the importance of assessing anxiety during pregnancy as part of suicide prevention strategies.
- Yang Y, Wang T, Wang D, et al. Gaps between current practice in perinatal depression screening and guideline recommendations: a systematic review. Gen Hosp Psychiatry. 2024.
  - ○ This systematic review identifies discrepancies between recommended screening practices and real-world implementation of perinatal mental health screening. The findings highlight structural barriers that may contribute to the under-detection of suicidal ideation in postpartum populations.

## Data Availability

No datasets were generated or analysed during the current study.
